# Pancreatic Cancer-Secreted Proteins: Targeting Their Functions in Tumor Microenvironment

**DOI:** 10.3390/cancers15194825

**Published:** 2023-10-01

**Authors:** Anna Lisa Cammarota, Antonia Falco, Anna Basile, Carlo Molino, Massimiliano Chetta, Gianni D’Angelo, Liberato Marzullo, Margot De Marco, Maria Caterina Turco, Alessandra Rosati

**Affiliations:** 1 Department of Medicine, Surgery and Dentistry “Schola Medica Salernitana”, University of Salerno, 84081 Baronissi, Italy; acammarota@unisa.it (A.L.C.); afalco@unisa.it (A.F.); abasile@unisa.it (A.B.); marzullo@unisa.it (L.M.); mcturco@unisa.it (M.C.T.); 2 General Surgery Unit, A.O.R.N. Cardarelli, 80131 Naples, Italy; carlo.molino@aocardarelli.it; 3 Medical and Laboratory Genetics Unit, A.O.R.N., Cardarelli, 80131 Naples, Italy; massimiliano.chetta@aocardarelli.it; 4 Department of Computer Science, University of Salerno, 84084 Fisciano, Italy; giadangelo@unisa.it; 5 FIBROSYS s.r.l., University of Salerno, 84081 Baronissi, Italy

**Keywords:** pancreatic ductal adenocarcinoma, secretome, cell signaling, tumor microenvironment, small molecules, monoclonal antibodies

## Abstract

**Simple Summary:**

Pancreatic ductal adenocarcinoma (PDAC) is predicted to become the second leading cause of cancer-related deaths by 2030. The main reasons for such a poor prognosis can be attributed to the particularly complex anatomical region in which the tumor grows, as well as the fact that this tumor is usually diagnosed at an advanced stage in most patients. At the molecular level, heterogeneous oncogenic and loss-of-function mutations in tumor suppressors, in which KRAS variants are the most frequent, characterize pancreatic cancer cells. Furthermore, altered ductal cells constitute only a modest portion of pancreatic cancer tumor mass, with the remainder made up of stromal cells and components. Indeed, the complex tumor microenvironment (TME) and communication between tumor and stromal cells are associated with different tumor cell phenotypes. In this context, transformed cells are the source of different extracellular signals that, when captured by nearby non-transformed stromal cells, influence tumor formation, metastasis, and even treatment efficacy. In this context, it is evident that this disease urgently requires a better knowledge of its biology in order to develop effective treatments. Here, we draw a special attention to pancreatic cancer-secreted proteins, which are primary players in the development and the maintenance of the “cancer-friendly” environment and reported, in this framework, druggable molecular targets for the design of more effective therapeutic treatments.

**Abstract:**

Pancreatic Ductal Adenocarcinoma (PDAC) is a ravaging disease with a poor prognosis, requiring a more detailed understanding of its biology to foster the development of effective therapies. The unsatisfactory results of treatments targeting cell proliferation and its related mechanisms suggest a shift in focus towards the inflammatory tumor microenvironment (TME). Here, we discuss the role of cancer-secreted proteins in the complex TME tumor-stroma crosstalk, shedding lights on druggable molecular targets for the development of innovative, safer and more efficient therapeutic strategies.

## 1. Pancreatic Cancer Fact Sheet

The most common type of pancreatic cancer is the Pancreatic Ductal Adenocarcinoma (PDAC), which arises from the ductal epithelium of the organ. Around 70% of pancreatic cancers begin in the organ’s head, with the majority starting from the ducts that transport digestive enzymes [1,2]. The prevalence rate is 49.8 cases per million people, while the predicted global incidence rate is 58.6 cases per million people annually. Annual mortality is projected to be 57.7 per million persons. Over the past 25 years, there has been a 55%, 63% and 53% increase of the incidence, prevalence and death rates, respectively. While representing only 1.8% of all malignancies, pancreatic cancer is responsible for 4.6% of cancer-related fatalities. Men have a somewhat greater incidence, prevalence, and fatality rate. By 2060, it is anticipated that pancreatic cancer deaths would have increased almost 1.97-fold [3].

Despite recent advances in surgical techniques and medical therapies, the median survival time for a pancreatic cancer patient at the time of diagnosis is 4–6 months, with a 12% five-year survival rate [3,4]. Pancreatic cancer is predicted to become the second leading cause of cancer-related deaths in the United States by 2030, surpassing colorectal, breast, and prostate cancer. Moreover, it has been reported that by 2040, gastrointestinal cancers (pancreatic, liver, and colorectal cancer) are expected to be three of the four leading causes of cancer death [5].

The main reasons for such a poor prognosis can be attributed to the particularly complex anatomical region in which the tumor grows, as well as the fact that this tumor is usually diagnosed at an advanced stage in most patients. 

The early stage and onset of the disease are characterized by rather vague disorders, which are often clinically misinterpreted, leading to delays in diagnosis until a stage marked by its fatal spread. The survival rate of patients dramatically drops in the first year after the diagnosis. Unfortunately, significant improvements in therapeutic protocols for this neoplasm have not been developed in the last three decades, resulting in a uniformly poor prognosis worldwide [5,6]. The only potentially curative treatment is surgical removal, but radical surgical resection of the tumor is indicated only in cases of intrapancreatic disease that does not extend to the retroperitoneum or transverse mesocolon and does not involve infiltration of the superior mesenteric artery, celiac tripod, or spleno-mesenteric-portal axis. For this reason, pancreatic cancer is only resectable in 10–20% of patients at the time of diagnosis, while it is locally advanced in 30–35% of patients due to infiltration of the large abdominal vessels, and metastases are already present in more than 50% of cases. However, even in the early stages, the prognosis is poor, with median survival rates with surgery alone in this group of patients being in the order of 12 months, and the five-year survival rate is between 5 and 10%. Median survival for stages III and IV is 10 and 6 months, respectively [7,8].

Unlike metastatic diseases in other tumor types (e.g., colon [9], gastroesophageal [10], head and neck [11], breast [12], lung [13] cancers) the first-line treatment for metastatic pancreatic cancer is still chemotherapy [14]. Indeed, even though immunotherapies and/or targeted therapies for the majority of solid tumors have advanced quickly, progresses in the treatment of pancreatic ductal adenocarcinoma have been unusually slow [15,16].

At the molecular level, the oncogenic mutations of the *KRAS* gene and loss-of-function mutations in tumor suppressors, such as *TP53*, *CDKN2A*, *SMAD4* and *BRCA2*, are the most frequent alterations in PDAC. *KRAS* mutations are the most frequent and are present in 88% of patients [17], with most of them characterized by the amino acid substitutions p.G12D (43%), p.G12V (31%), p.G12R (14%) and p.G12C (1.9%) [18,19]. The development of allele-specific (targeting *KRAS* p.G12C and *KRAS* p.G12D) or a broader pan-RAS class of small molecule inhibitors has stimulated substantial interest in treating *KRAS* mutated malignancies, including PDAC [20]. The efficacy of the first p.G12C *KRAS* inhibitor approved by the FDA was studied in PDAC patients and showed a temporary response in only about 21% of patients [21]. This limited response was previously observed in lung cancer patients and was characterized by immunological evasion through tumor cell-intrinsic and non-cell autonomous pathways inducing drug resistance [22]. It is probable that similar problems will be faced when testing further *KRAS* inhibitors if they ever reach the clinic. Notably, a different approach, based on a first-in-class *KRAS* p.G12D selective degrader (ASP3082) is currently being evaluated in pancreatic cancer patients [23]. However, an additional challenge in this scenario comes from intra-tumor heterogeneity, which is defined by the presence of multiple genetic drivers in the same tumor and thwarts efforts in precision medicine [24]. In this regard, a pilot clinical research recently indicated that genomically matched targeted drug combinations were active in advanced pancreatic tumors [25]. 

Additionally, mutated epithelial, acinar, or ductal cells account for a minor fraction of pancreatic cancer tumor mass, with the remainder made up of stromal cells and components. Indeed, the complex tumor microenvironment (TME), as well as communication between tumor and stromal cells, are associated with extremely diverse tumor cell phenotypes that are also highly adaptable through the formation of metabolic niches [26]. Individual clones collaborate to fine-tune components of the tumor micro-environment that promote a fitness advantage for tumor proliferation and immune evasion, thus driving PDAC progression [27]. The particular immunosuppressive micro-environment of pancreatic cancer is formed by the accumulation of desmoplastic stroma and the infiltration of immunosuppressive cells such as myeloid suppressor cells (MDSCs), tumor-associated macrophages (TAMs), cancer-associated fibroblasts (CAFs), regulatory T cells (Tregs), and tumor-associated cytokines, posing a challenge for immunotherapy. Few clinical trials of immunotherapeutics, aimed at re-tuning the TME against the tumor, have improved PDAC patient survival rates beyond the limited performances of current chemotherapy [28].

In this entangled maze of altered morphology and derailed cellular and molecular functions, transformed cells are the source of distinct extracellular signals [29] that, when captured by neighboring non-transformed stromal cells, influence tumor development, metastasis, and even drug efficacy [30]. In this field of study, researchers have recently discovered novel players in the TME, which can improve therapeutic approaches for several cancers [31]. Here, we draw special attention to pancreatic cancer-secreted proteins, which are primary players in the development and the maintenance of the “cancer-friendly” environment in the neoplastic lesion.

## 2. Proteins Secreted by Pancreatic Cancer Cells: Messages Sent to the Neighborhood

Cell communication in multicellular organisms allows cells to adapt their phenotypes and function. A number of secreted factors, whether soluble or associated with membranes, mediate critical molecular mechanisms involved in tissue and organism homeostasis. Typically, proteins follow the conventional protein secretion pathway, which involves the endoplasmic reticulum (ER) and the Golgi complex. However, some proteins use alternative routes, such as Unconventional Protein Secretion (UPS) pathways, induced by cellular stress such as nutrient deficiency, mechanical stress, inflammation, and ER stress. When the pathways leading to protein secretion, mediating both short- and long-range signals, are dysregulated, it accelerates disease pathogenesis. In addition, for some cancers, there is growing interest in intracellular proteins that, if secreted, play distinct functions, demonstrating that UPS pathways are still not fully understood [32]. In this context, tumor secretomes are able to influence the behavior of both neoplastic and non- neoplastic cells, providing a promising source of potential biomarkers useful in patient management. In fact, the alteration of the secretome mirrors disrupted cell-cell signaling in the pancreatic cancer milieu and participated in the reshaping of a fibrotic and inflammatory micro-environment that promotes cancer development and progression [33].

A literature search allowed us to compile a comprehensive dataset that could illustrate the main biochemical pathway involved in such a dramatic tissue transformation driven by pancreatic cancer cells. We used PubMed to search for reports and data on PDAC secretome studies. To maximize search specificity and sensitivity, the following keywords were used: “Pancreatic cancer”, “Pancreatic ductal adenocarcinoma”, “secretome, extracellular protein and pancreatic carcinoma” and “tumor microenvironment”. Only studies involving pancreatic cancer-secreted proteins were included after further screening based on title and abstract. There were no restrictions on the type of study. Manuscripts selected for further considerations primarily employ two methods for analyzing pancreatic cancer cells’ secretome: mass spectrometry and the Enzyme-Linked ImmunoSorbent Assay (ELISA). Most mass spectrometry-based studies include secretome analysis of human [34,35,36,37,38,39], murine [40] or hamster [41] cell lines described as relevant preclinical models of pancreatic cancer [42]. On the other hand, most studies were carried out using ELISA methods on sera of patients with hereditary predisposition [43] as well as with advanced disease [44,45], and on pancreatic cancer cell culture supernatant [35]. In addition, we included a recent review [46] resuming the use of novel protein biomarkers in early PDAC diagnosis, prognosis and treatment response prediction. Finally, an approach integrating meta-analysis of PDAC secretome MS data, used to identify potential disease biomarkers [47], was also considered (Table 1).

**Figure 1:**
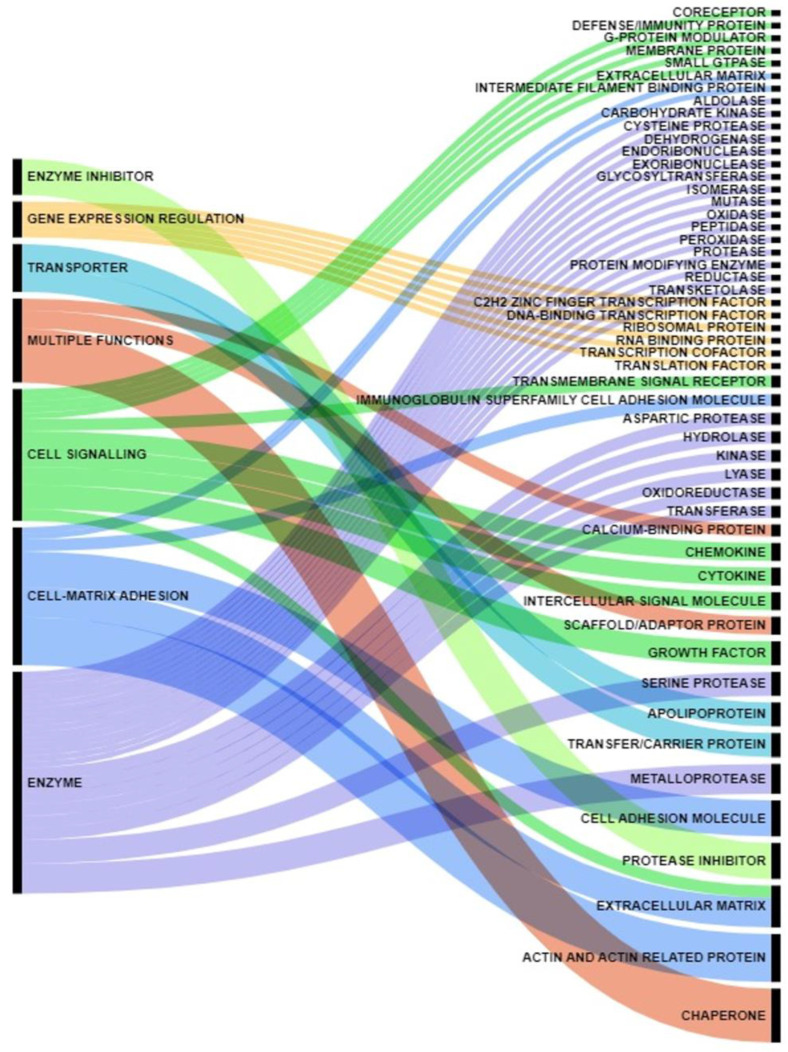
Proteins Functions—A Global Core Biodata Resource, Panther Classification System (https://pantherdb.org/, accessed on 11 June 2023), classified the gene dataset for functions. Proteins secreted by pancreatic cancer cells were classified as having a general function (left column) or a specific function (right column). An Alluvial plot (https://www.mapequation.org/alluvial/, accessed on 28 June 2023) was used to depict the classifications. Furthermore, percentages of secreted proteins having common general functions are reported in the left column. A color code was used for each general function. In the column, the most represented protein functions are in the plot’s lower part.

The obtained dataset represented and summarized the coexistence of cytokines, growth factors, extracellular matrix proteins, proteases and protease inhibitors, membrane and extracellular vesicle-associated proteins, and metabolic enzymes in the neoplastic milieu (Figure 1). Enzymes accounted for 32% of the proteins reported in the study, whereas enzyme inhibitors accounted for 5%. Cell signaling molecules accounted for 19%, while specific cell-matrix adhesion molecules accounted for 20%. Proteins with multiple roles accounted for 19% of the studied dataset, with the remaining 5% representing gene expression regulators and 6% representing transporters. Nonetheless, specific involvement in processes regulating tumor mass formation and development remains undisclosed for most of the proteins reported here.

To define protein clusters associated with main cellular processes, the dataset was analyzed in the GO biological processes 2023 database using Enrichr (https://appyters.maayanlab.cloud/, accessed on 7 June 2023) (Table 2). Proteins with known functions when secreted are then briefly discussed.

As a result of the analysis, it was possible to highlight a set of proteases responsible for extracellular matrix and cellular component disassembly. As previously reported, the acellular components of the pancreatic tumor mass, as well as their changes over time, drive the tumor’s progression. In this regard, MMP-2 and MMP-9 (Matrix MetalloProteinases) gelatinases are abnormally and contemporarily upregulated in pancreatic cancer [48], but the clinical relevance measured by the correlation between their expression and survival, metastasis, or tumor stage is debatable [49]. Instead, the expression of the matrilysin MMP-7 in tumor samples was linked to a poorer prognosis in patients [50] and an unfavorable pathologic response to neoadjuvant therapy [51]. Furthermore, the multifunctional zinc finger transcription factor YY1 (Yin Yang 1) has been shown to suppress stromelysin-2 (MMP-10), whose overexpression is a favorable independent prognostic factor in PDAC patients [52]. Finally, the secreted gelsolin, a scavenger of extracellular actin, has been recently reported for its involvement in attenuating DNGR-1-dependent dendritic cell-mediated anti-tumor immunity [53].

Some apoptosis-regulating proteins with very different biochemical functions have also been identified, and some of them have been linked to a role in pancreatic cancer. For example, Hsp90AA1 (Heat Shock Protein 90 Alpha Family Class A Member 1) is one of the most abundant proteins expressed in cells, interacting with several secreted client proteins. Hsp90AA1 promotes tumor aggressiveness and chemoresistance by activating AKT through LRP-1 (Low-density lipoprotein Receptor-related Protein 1) [54]. Among other chaperones, PARK7 (Parkinson protein 7) [55] and PPIA (Peptidylprolyl Isomerase A) [42] are upregulated and secreted by cancer cells. While PARK7 has been described for its ability to counteract environmental oxidative stress [56], PPIA is known to act through the CD47 and NF-kB pathway, thus promoting cell proliferation [57]. In addition, the extracellular chaperone Clusterin (CLU) has been shown to be a mediator of chemoresistance in pancreatic cancer [58]. The overexpression of the co-chaperone BAG3 (BCL2 Associated Athanogene 3) has also been described as associated with pancreatic cancer aggressiveness [59], and its sera levels are measurable in pancreatic cancer patients [45,60]. Furthermore, a distinct function was described for secreted BAG3. Indeed, its interaction with IFITM2 (Interferon Induced Transmembrane Protein 2) on the plasma membrane can induce pro-tumoral cytokine release by macrophages [61] and fibroblasts [62], thus sustaining tumor growth. Another relevant protein in this context is the soluble GSTP1 (Glutathione S-Transferase Pi 1), able to catalyze the conjugation of many hydrophobic and electrophilic compounds with reduced glutathione. This activity plays a role in regulating oxidative stress, thus negatively affecting proliferation and upregulating apoptosis in PDAC cells, as demonstrated by knockdown experiments [63]. The transglutaminase-2 gene (TGM2) has also been identified as an important extracellular cross-linking enzyme involved in ECM turnover, and its levels were associated with poor survival in pancreatic cancer patients [64]. Annexins dysregulation has also been reported as a common feature in several cancers [65], and, among them, soluble ANXA5 (Annexin A5) has been described for its activity in cell membrane resealing [66], an essential repair machinery that promotes survival in invasive cancer cells [67]. The insulin-like growth factor (IGF)-axis, which mediates survival signals essential for pancreatic cancer development and progression, belongs to traditional signaling molecules, and its crucial role was evidenced by the strong association between SNPs (Single Nucleotide Polymorphisms) in correlated IGF1R, IGF2R, and IRS1 genes with tumor response to therapy and disease stage [68]. Finally, AXL (a member of the TAM tyrosine kinase receptors) is present in our dataset. The AXL/Gas6 (Growth Arrest Specific 6) signaling pathway plays a role in tumor cell proliferation, and plasma detection of the AXL soluble form, obtained by an ADAM10/ADAM17 (ADAM Metallopeptidase Domain)-specific shedding mechanism, has been described as an early signature of PDAC [69], while its functions, likely relying on attenuating Gas6 functions in the TME, remain unknown [70].

An additional functional cluster of secreted proteins was linked to neutrophil chemotaxis. Tumor-infiltrating neutrophils indicate a poor prognosis for patients, and activated neutrophils can generate neutrophil extracellular traps (NETs), which are emerging in several cancers as markers of cancer progression and immunosuppression [71,72]. As a first example, extracellular Galectin-3 (LGALS3), detected in the blood of PDAC patients [73], has been associated with neutrophils recruitment and inflammation exacerbation in several infectious diseases [74]. Indeed, given the similarities in the pathogenesis of inflammatory diseases and cancer, it is not surprising to find in the described pancreatic cancer-secreted proteins dataset molecules such as IL8 (CXCL8) [75], Eotaxin-2 (CCL24) [76], PPBP (Pro-Platelet Basic Protein) [77], and PF4 (Platelet Factor 4) [78], which are potent neutrophil chemotactic factors. Serum Amyloid A1 (SAA1) [79], another protein induced in pancreatic cancer cells, attracts neutrophils to the tumor nest by interacting with TLR2 (Toll-Like Receptor 2) [80].

Moreover, altered carbohydrate catabolism has been recognized as the major metabolic alteration in pancreatic cancer [81], but the role of those enzymes in the pancreatic cancer milieu has not been fully elucidated yet. The evidence that secretory PKM (Pyruvate Kinase M1/2) promotes tumor angiogenesis by facilitating endothelial cell proliferation and new vessel formation via the PI3K/AKT and Wnt/-catenin signaling pathways provides some hints [82]. On the other hand, secreted PGK1 (Phosphoglycerate Kinase 1) has been shown to act on angiostatin levels, resulting in an anti-angiogenic and tumor suppressive function [83]. Finally, enolases, specifically the cell surface- associated ENO1, have been identified as a pancreatic cancer neoantigen, promoting cancer cell survival and migration by coordinating with integrins and uPAR (Plasminogen Activator, Urokinase Receptor) [84].

Other proteins relevant in this context, but not included in Table 2, are: GDF15 (Growth Differentiation Factor 15), whose expression was positively correlated with poor survival in PDAC patients and whose downregulation inhibited PDAC tumor growth in vivo [85]; LIF (LIF Interleukin 6 Family Cytokine), a pleiotropic cytokine that regulates cell survival by interacting with its receptor LIFR/GP130 expressed on surrounding fibroblasts, promotes a pro-invasive phenotype [86]; VEGF (Vascular Endothelial Growth Factor) [87], which functions as an endothelial cell mitogen and is strongly linked to angiogenesis in pancreatic cancer; and TF (Tissue Factor), which, in its alternatively spliced form asTF, is released by cancer cells and triggers the activation of PI3K/Akt, MAPK, and FAK pathways through its interactions with α6β1 and αvβ3 integrins [88].

The whole analysis highlighted the presence of major cellular processes relevant in the pancreatic tumor TME.

## 3. Targeting Pancreatic Cancer-Secreted Proteins

The list of proteins resulting from the analysis was used to screen the presence of pharmacologic molecules designed to interfere with their activity, describing available candidates for therapeutic use in pancreatic cancer.

### 3.1. Communications Breakdown Operated by Small Molecule Drugs

The DGIdb (The Drug Gene Interaction Database, accessed on 28 June 2023) was queried, screened and integrated with a literature search for available molecules possibly having inhibitory activity on pancreatic cancer-secreted proteins illustrated above; the obtained search results are described below.

The response to synthetic inhibitors of MMPs (MMPIs) was studied in the past decades in several solid tumors. However, despite promising preclinical data, all trials were unsuccessful in reducing tumor mass or improving overall survival [89].

Clusterin expression was challenged using the drug OGX-011, an antisense oligonucleotide that showed a potentiating effect on various FDA-approved anticancer chemotherapeutics during clinical trials [90]; however, no trial in pancreatic cancer has been programmed yet [91].

Ganetespib (STA-9090) is a small molecule that interferes with HSP90 client protein binding, thus promoting the inactivation and degradation of the signaling proteins that regulate cancer progression. Unfortunately, a Phase II study carried out in refractory metastatic pancreatic cancer failed to prove its clinical efficacy [92]. More clinical trials as a neoadjuvant treatment and/or in combination with chemotherapy are expected [93].

The extracellular galectin-3 functions were shown to be targeted by RN1, an arabinogalactan polysaccharide isolated from the flowers of the Chinese ginseng plant (Panax notoginseng), which displayed antitumoral activity in PDAC in vitro and in vivo [94]. While the use of galectin inhibitors, such as belapectin (GR-MD-02), has been accepted as a novel therapeutic tool in liver antifibrotic therapy [95], clinical trials are ongoing to explore its utility in lung, head and neck cancer diseases (NCT02575404).

### 3.2. Communications Breakdown Operated by Monoclonal Antibodies

The target specificity of monoclonal antibodies (mAbs) makes them powerful tools for a wide spectrum of biomedical and clinical application. As previously stated, the use of DGIdb was supported and integrated by a literature search to identify available mAbs able to bind and neutralize the secreted proteins here selected for discussion.

Xentuzumab, an IgG1 monoclonal antibody with high affinity for IGF-1 and IGF-2 currently tested in preclinical models for the treatment of advanced solid tumors, allowed the collection of several interesting data [96,97]. A phase I trial enrolling patients affected by different advanced solid tumors, including PDAC, allowed verifying its safety, tolerability, and clinical manageability. On the other hand, in a phase II study in metastatic breast cancer, treatments with Xentuzumab combined with everolimus and exemestane did not show a significant impact on PFS (Progression Free Survival) [98].

Another strategy, aimed at neutralizing VEGF with the monoclonal antibody Bevacizumab, showed promising results in preclinical studies [99], but it did not show appreciable benefits when combined with gemcitabine in clinical trials [100]. Thanks to its high safety profile, trials were further extended to a third compound, erlotinib, but still without satisfying results [101].

Recently, LIF, which seems to directly fuel oncogenic *KRAS* signaling, has been proposed as a therapeutic target in PDAC, thus providing a chance to develop a new tool able to overcome chemotherapy resistance in *KRAS* targeted protocols. Indeed, in syngeneic mouse models of oncogenic Kras p.G12D driven pancreatic adenocarcinoma, using LIF neutralizing antibodies, or only gemcitabine, had no effect on tumor growth, while the combined treatment was able to repress tumor growth and improve animal survival [102]. A recently completed first-in-human trial with a humanized anti-LIF mAb (MSC-1) in advanced solid tumors showed that the mAb was well-tolerated and was able to extend the PFS of some patients [103]; a phase II study in PDAC patients is currently underway (D8151C00001).

In this context, the neutralization of extracellular BAG3 is another promising strategy supported by studies carried out in several murine preclinical models treated with an anti-BAG3 mAb, which showed its ability in reducing PDAC tumor growth as monotherapy [61,104]. But even more striking results were observed in combined treatments with the ICIs (Immune Check-point Inhibitors) anti-PD-1 [105] and anti-SIRP-alpha [106]. Ex vivo studies on mouse tissues showed that combo treatments were able to promote an immunostimulatory microenvironment along with a significant reduction of cancer fibrosis [105,106,107]. Further refinements and developments of the anti-BAG3 mAb-based therapies could hopefully contribute additional evidence of efficacy and safety of combined treatments and could allow tailoring and diversify the protocols for other solid tumors [108].

An anti-IL-8 antibody was also tested in a humanized mouse model of PDAC in combination with anti-PD-1. The treatment resulted in significantly reduced tumor growth, as well as an increased innate immune response and type I cytokine expression in myeloid cells, revealing a novel function of the IL-8 antibody in myeloid cell “re-education” [109]. HuMax-IL8 was tested in a Phase I trial on solid tumors, showing its safety and tolerability, while further studies are ongoing to evaluate the efficacy of anti-IL-8 treatments combined with other immunotherapies [110].

**Figure 2:**
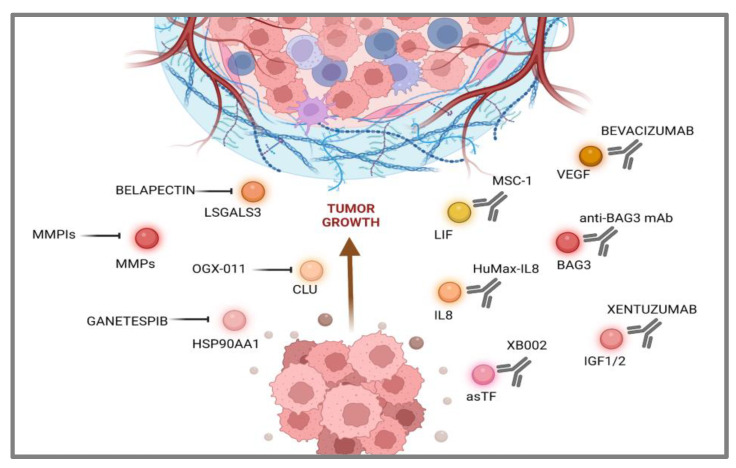
Druggable secreted proteins landscape in pancreatic cancer cells. Inhibition arrows were used for small molecule drugs. Image was realized using Biorender (https://app.biorender.com/, accessed on 7 June 2023).

Another strategic perspective aims at targeting the asTF protein by a first-in-class humanized antibody, which exerted a significant effect on tumor growth in an animal model, downregulating several gene function categories, including focal adhesion, cell motility, cell proliferation, cytoskeleton, regulatory proteases and cell death, many of which are known to be TF- associated [111]. In this case, XB002, a novel, investigational ADC (Antibody Drug Conjugate), is currently being tested as a single-agent and combination therapy in subjects with inoperable locally advanced, or metastatic solid tumors in a Phase I trial; results are expected in late 2024 (NCT04925284) (Figure 2).

## 4. Conclusions and Future Directions

The abnormal production of secreted factors in malignant cells, via canonical and non-canonical pathways, has long been the key mechanism through which metabolic rewiring of cancer cells and neighboring non-malignant cells directs tumor progression [112]. With a focus on targeting pancreatic cancer-secreted proteins, we tried to summarize current and hopefully promising novel therapeutic approaches for the treatment of pancreatic cancer. The study, carried out by analyzing the data available in the literature, confirms the need for further efforts in selecting new molecular targets with lower toxicity risks along with the design of more appropriate, selective and specific therapeutic tools. Furthermore, deep profiling of the tumor tissue proteome, as well as circulating proteins, using high-throughput technologies during the pre-treatment stage could help tailor personalized therapies. Indeed, further addressing the heterogeneity in cancer cells, stroma, and immune cells may lead to the discovery of biomarkers panels that may aid in the selection of a small proportion of patients who could benefit from more effective therapeutic strategies for this fatal disease.

## Figures and Tables

**Table 1:** Pancreatic cancer cells-secreted proteins and pancreatic cancer patient’s sera proteins dataset.

Gene Names	References
**Pancreatic cancer cells-secreted proteins**	
*LGALS1, ENO2, SERPINA1, NMI, PRDX4*	Chung J.C. et al. 2008 [34]
*CP, LGALS3, MARCKS*	Brandi J. et al. 2016 [35]
*GDF15, TGM2, LIF, MMP2*	Li X. et al. 2022 [36]
*SAA1, RC3H1, CCT8, ZNF518B, EXOSC8, IGF2, NPC2, HSP90AA1, PPIA, ENO1,*	Liu P. et al. 2019 [37]
*DNAJB11, PPT1, CTSD, CDH3, PLAU, LFNG*	Liu P. et al. 2016 [38]
*GLRX3*	Jo J.H. et al. 2021 [39]
*PLEC*	Kelly K.A. et al. 2008 [40]
*MMP12, MMP10, LAMA5, WHAG, CPN1, THPH2*	Liu P. et al. 2020 [41]
*ALB, ENO1, FN1, TF, LGALS1, APOE, CTSD, TPI1, GSTP1, PARK7, PRSS1, MSN, PGK1, ANXA5, PIN1, PKM, EEF1A1, THBS1, GSN, LGALS3, TIMP1, CFL1, FLNA, LGALS3BP, CALR, CLIC1, TAGLN2, LDHA, NME1, TKT, SFN, ALDOA, ENO2, PGAM1, ARHGDIA, ACTB, P4HB, ACTA1, AHSG*	de Oliveira G. et al. 2020 [47]
**Pancreatic cancer patients’ sera proteins**	
*CXCL8, LCN2, MUC5AC*	Levink J.M. et al. 2022 [43]
*ULBP2, NAPA, TGFBI, RAB14, ULBP2, CP, RPL22, PURB, C1S, ANXA11, ERO1L*	Chang Y.T. et al. 2011 [44]
*ALCAM, ANG, AXL, BAG3, BSG, CCL24, CEACAM5, CEACAM1, CLU, COL18A1, EPCAM, HP, ICAM1, IGFBP2, IGFBP4, LCN2, LRG1, MMP2, MMP7, MMP9, MSLN, PARK7, PF4, PPBP, PRG4, SPARCL1, SPP1, TGFBI, THBS1, TIMP1, TNFRSF1A, VEGF*	Firpo M.A. et al. 2023 [45]
*THBS2, IGFBP2, IGF1, ENPP2, LRG1, TTR, APOE, ITIH3, APOA1, APOL1, TFF1, TFF2, TFF3, GDF15*	Kapszewicz M. et al. 2021 [46]

**Table 2:** Clustered functions for pancreatic cancer-secreted proteins—Enrichr-Appyter online applications generated a downloadable table. The names of the genes were reported along with the *p*-values and q-values of significant terms in the chosen library. The q-value is an adjusted *p*-value calculated using the Benjamini-Hochberg method for multiple hypothesis testing correction.

Term	*p*-Value	q-Value	Overlaps Genes
Extracellular Matrix Disassembly (GO:0022617)	7.05 × 10^−9^	2.09 × 10^−6^	*[MMP12, PRSS1, GSN, MMP7, MMP2, MMP9, MMP10]*
Regulation of Apoptotic Process (GO:0042981)	2.42 × 10^−11^	3.59 × 10^−8^	*[HSP90AA1, GSTP1, ANXA5, PARK7, IGF1, CLU, MMP9, THBS1, ACTB, NME1, LGALS1, AXL, BAG3, CEACAM5, ARHGDIA, CFL1, ALB, PPT1, FLNA, CALR, PPIA, CTSD, TGM2]*
Neutrophil Chemotaxis (GO:0030593)	1.94 × 10^−7^	2.88 × 10^−5^	*[LGALS3, CCL24, CXCL8, SAA1, PPBP, PPIA, PF4]*
Carbohydrate Catabolic Process (GO:0016052)	4.19 × 10^−9^	1.56 × 10^−6^	*[LDHA, TPI1, PKM, PGAM1, PGK1, ENO1, ENO2]*

## Data Availability

The data that support the findings of this study are available on request from the corresponding authors (M.D.M and A.R.).
